# Prevalence of Fatigue Among Inflammatory Bowel Disease Patients at a Tertiary Center in Saudi Arabia

**DOI:** 10.3390/jcm15082941

**Published:** 2026-04-13

**Authors:** Mariam S. Mukhtar, Mahmoud Mosli, Nadeem Butt, Saud M. Bamousa, Sharefah A. Alqarni, Mohammad Mustafa, Yasser Bawazir, Roaa Alsolaimani

**Affiliations:** 1 Department of Internal Medicine, Faculty of Medicine, King Abdulaziz University, Sulimaniah Street 059, Jeddah 21589, Saudi Arabia; 2 Inflammatory Bowel Disease Unit, King Abdulaziz University Hospital, Jeddah 21589, Saudi Arabia; 3 Department of Family and Community Medicine, Faculty of Medicine Rabigh, King Abdulaziz University, Jeddah 21589, Saudi Arabia; 4 Internal Medicine Department, King Abdulaziz University Hospital, Jeddah 21589, Saudi Arabia; 5 First Health Cluster, King Abdulaziz Hospital, Jeddah 21589, Saudi Arabia; 6 Department of Medicine, Medical College, University of Jeddah, Jeddah 21589, Saudi Arabia; mamustafa@uj.edu.sa

**Keywords:** inflammatory bowel disease, fatigue, Brief Fatigue Inventory (Arabic), Saudi Arabia, prevalence, biopsychosocial factors

## Abstract

**Background:** Fatigue is a common and distressing symptom in inflammatory bowel disease (IBD), yet it is rarely addressed in routine care. Most available evidence comes from Western and East Asian populations, with limited data from the Middle East. **Objectives:** To estimate the prevalence of fatigue in Saudi patients with IBD, using the Arabic-validated Brief Fatigue Inventory (BFI-A), and to examine associations with demographic, clinical, treatment, and laboratory factors. **Methods:** This cross-sectional study was conducted at King Abdulaziz University Hospital, Saudi Arabia, between March and December 2025. Patients aged ≥12 years with histologically confirmed IBD completed a structured telephone interview. Demographic characteristics, comorbidities, IBD control scores, Montreal classification, medication history, and laboratory results were collected. Patients experiencing severe flares, hospitalization, or another primary condition likely to explain fatigue were excluded. Fatigue severity was classified as none, mild, moderate, or severe. Associations were tested using chi-square and Kruskal–Wallis tests. **Results:** Among 286 patients (mean age, 30.8 ± 9.1 years; 57.7% male), 23.1% reported mild fatigue, 36.4% moderate fatigue, and 19.2% severe fatigue on the BFI-A. Fatigue severity was not associated with demographic factors, IBD type or phenotype, treatment exposure, or most laboratory parameters. Only serum iron (*p* = 0.011) and erythrocyte sedimentation rate (*p* = 0.023) differed across fatigue categories, without a clear dose–response pattern. **Conclusions:** Fatigue affects more than half of Saudi patients with IBD and is not explained by routine clinical or laboratory factors. Routine fatigue assessment and attention to biopsychosocial contributors may improve IBD care.

## 1. Introduction

Inflammatory bowel disease (IBD), including Crohn’s disease (CD) and ulcerative colitis (UC), is a chronic, immune-mediated condition characterized by relapsing and remitting gastrointestinal inflammation [1,2]. Alongside symptoms such as abdominal pain, diarrhea, rectal bleeding, and weight loss, many patients experience fatigue that can substantially limit daily functioning and well-being [1,2,3]. Systematic reviews and cohort studies consistently show that fatigue is more frequent and more severe in IBD than in the general population and is not necessarily related to active inflammation [3,4,5,6,7,8]. Fatigue also has a marked impact on health-related quality of life (HRQoL), social and family roles, and work productivity; patients often describe it as one of the most debilitating aspects of their disease [3,5,6,8,9].

Although there is no single agreed-upon definition, fatigue is commonly described as an overwhelming, persistent sense of tiredness or lack of energy that is disproportionate to recent activity and not fully relieved by sleep or rest [10,11]. In IBD, fatigue is increasingly recognized as a multidimensional problem with physical, emotional, and cognitive components [8,10,11,12,13]. Physical fatigue limits day-to-day activity; emotional fatigue is associated with low motivation and mood changes; and cognitive fatigue manifests as reduced concentration, memory difficulties, and impaired emotion regulation [10,11,12,13].

The mechanisms underlying IBD-related fatigue are complex and multifactorial. Proposed contributors include persistent systemic and intestinal inflammation, cytokine-mediated effects on the central nervous system, anemia and iron deficiency, endocrine abnormalities, medication side effects, sleep disturbance, and comorbid mood disorders [7,8,12,13,14]. Recent reviews suggest that inflammatory and non-inflammatory pathways are closely interlinked, with disease activity and its treatments influencing fatigue both directly through inflammation and indirectly via effects on mood, sleep, pain, and physical activity [7,8,10,12,13,14].

Across Europe, North America, and East Asia, epidemiological studies typically report clinically relevant fatigue in approximately 40–60% of adults with IBD overall, rising to 70–80% among those with active disease [4,5,6,7,8,15,16,17,18,19,20,21,22]. Even in patients experiencing clinical or biochemical remission, around 30–50% continue to report fatigue. In several cohorts, depressive and anxiety symptoms and sleep problems show stronger and more consistent associations with fatigue than hemoglobin levels or conventional inflammatory markers [7,8,13,15,16,17,18,19,20,21,22]. More detailed studies from Spain, Scandinavia, East Asia, and Mexico, including multidimensional fatigue measures and remission-focused cohorts, reinforce the same message: fatigue is common, often persistent, and only partly explained by disease activity and standard laboratory tests [13,15,16,17,18,19,20,21,22,23,24,25].

In contrast, data from the Middle East remain sparse. A recent review of HRQoL and fatigue in IBD highlighted the lack of studies from this region, including Saudi Arabia [3,26]. The few available studies are small, single-center, and limited in clinical and laboratory characterization. Moreover, only a minority have used dedicated Arabic-language fatigue instruments that are translated and validated, such as the Brief Fatigue Inventory–Arabic (BFI-A) [27]. Cultural factors, including differences in healthcare-seeking behaviour, stigma around mental health, and reporting norms, may also influence how fatigue is expressed and communicated in Arab populations. These cultural dimensions remain largely unexplored in the IBD fatigue literature and are an important consideration when interpreting our findings.

To address these gaps, we conducted a cross-sectional study at a tertiary IBD center in western Saudi Arabia. Our primary aim was to describe the prevalence and severity of fatigue in Saudi patients with IBD using the BFI-A [27]. Our secondary aim was to examine how fatigue severity relates to demographic factors, IBD type and phenotype, treatment history, and routinely measured laboratory parameters. By comparing our findings with international data, we aimed to provide context-specific evidence on the burden and correlates of fatigue in IBD in the Middle East [2,3,4,7,8,26].

## 2. Materials and Methods

### 2.1. Study Design and Setting

This cross-sectional observational study was conducted between March and December 2025 at King Abdulaziz University Hospital, a tertiary hospital in Saudi Arabia. Using the hospital’s electronic records, we identified patients aged ≥12 years with a histologically confirmed diagnosis of IBD.

Ethical approval was obtained (Unit of Biomedical Ethics, Research ethics Committee (REC), NCBE Registration number HA-02-J-008, King Abdulaziz University). Participation implied consent. All procedures were conducted in accordance with the principles of the Declaration of Helsinki.

### 2.2. Participants and Data Collection

Eligible patients completed a structured telephone interview that captured demographic characteristics, smoking status, comorbidities, and fatigue. Perceived disease control was assessed using the Inflammatory Bowel Disease Control (IBD-Control) Questionnaire [28], which is routinely completed during clinic visits and was extracted from the electronic medical record.

Clinical IBD data were obtained from medical records, including diagnosis (CD, UC, or IBD-unclassified) and disease phenotype based on the Montreal classification (age at diagnosis; location and behavior; presence of perianal disease for CD; and extent and severity for UC) [29]. Medication history was recorded, focusing on exposure to 5-aminosalicylates, systemic corticosteroids (including route and clinical response), and biologic agents. For patients with UC, disease activity was assessed using the Mayo score. A formal validated activity index (e.g., Harvey-Bradshaw Index or Crohn’s Disease Activity Index) was not systematically applied for CD patients in this study. Although endoscopic and radiological assessments were available for some patients as part of routine care, they were not consistently performed contemporaneously with the fatigue assessment across the full CD cohort. This represents a limitation that is acknowledged in Section 4.5.

We also collected recent laboratory results, including complete blood count (CBC), erythrocyte sedimentation rate (ESR), C-reactive protein (CRP), serum iron, ferritin, transferrin saturation, thyroid-stimulating hormone (TSH), and serum 25-hydroxyvitamin D. For each patient, the laboratory values closest to the fatigue assessment within a predefined time window were used. For CRP, a value of >5 mg/L was used as the threshold for elevated levels, consistent with the laboratory reference range at our institution.

Based on chart review and clinical judgment, we excluded patients with a severe IBD flare, those currently hospitalized, or those with another medical condition likely to explain fatigue, such as severe anemia, active malignancy, or a neuromuscular disorder [4,6,8,12]. For the purposes of this study, a “severe IBD flare” was defined as a clinical presentation requiring hospitalization or urgent escalation of therapy (including intravenous corticosteroids or biologic induction) at the time of assessment. For UC, this broadly corresponded to a Mayo score of S3 with systemic features; for CD, it referred to patients with frank clinical deterioration necessitating inpatient care or emergency intervention. Patients who had stable, chronic symptom burdens—including those with moderate disease activity—were eligible for inclusion, as fatigue is recognized across the full spectrum of disease activity.

### 2.3. Fatigue Assessment

Fatigue was measured using the BFI-A, the validated Arabic-language version of the Brief Fatigue Inventory [27]. The instrument was administered verbally during a telephone interview. In line with our prespecified protocol and prior use of the BFI/BFI-A in chronic disease settings [10,27], we categorized BFI-A scores into four levels: no fatigue, mild fatigue, moderate fatigue, and severe fatigue. Fatigue categories were defined as follows based on BFI-A mean scores: no fatigue (score = 0), mild fatigue (score >0 to ≤3), moderate fatigue (score >3 to ≤6), and severe fatigue (score >6 to ≤10). These thresholds are consistent with validated cut-points used in chronic disease populations. Mild fatigue may be associated with occasional tiredness that does not significantly interfere with daily activities; moderate fatigue implies meaningful interference with activity, mood, and work; and severe fatigue represents a high degree of exhaustion with substantial impact across multiple functional domains assessed by the BFI-A.

### 2.4. Statistical Analysis

Descriptive statistics were employed to outline the characteristics of the patients. Continuous variables were presented as either mean (standard deviation [SD]) or median (interquartile range [IQR]), depending on suitability, while categorical variables were expressed as frequencies and percentages. To compare across different fatigue levels (none, mild, moderate, severe), the Kruskal–Wallis test was utilized for continuous variables, and the chi-square test or Fisher’s exact test was applied for categorical variables, as appropriate. A two-sided *p*-value of less than 0.05 was considered statistically significant. All analyses were conducted using standard statistical software. Beyond univariate analyses, a multivariable logistic regression model was employed to determine independent predictors of moderate-to-severe fatigue (BFI-A categories). Fatigue severity was categorized into none/mild versus moderate/severe. Covariates were pre-selected based on clinical importance and included age, sex, comorbidity status, prior 5-ASA exposure, serum iron, ESR, and vitamin D level. Adjusted odds ratios (ORs) with 95% confidence intervals (CIs) were provided. Model discrimination was evaluated using the area under the receiver operating characteristic curve (AUC), and model fit was assessed using the Akaike information criterion (AIC) and Pseudo R^2^.

### 2.5. Manuscript Writing

The use of artificial intelligence was limited to minor language editing of the manuscript; all edits were reviewed and approved by the authors.

## 3. Results

### 3.1. Baseline Demographic and Clinical Characteristics

A total of 286 patients met the study’s inclusion criteria. Baseline characteristics are presented in Table 1. The mean (SD) age was 30.8 (9.1) years (range, 12–66 years), and 165 patients (57.7%) were male. Mean BMI was 23.8 (6.0) kg/m^2^. Most patients were non-smokers (244/286; 85.3%), with 32 (11.2%) current smokers and 10 (3.5%) former smokers.

Comorbidities were uncommon. Among 278 patients with available data, 40 (14.4%) had at least one comorbid condition, and 238 (85.6%) had none. The most frequent comorbidities were thyroid disease (7/270; 2.6%), hypertension (5/270; 1.9%), diabetes mellitus (3/270; 1.1%), and connective tissue disease (3/270; 1.1%).

Regarding IBD subtype, 184 patients (64.3%) had CD, 94 (32.9%) had UC, and 8 (2.8%) had IBD-unclassified (Table 1). Among patients with CD, most were diagnosed between 17 and 40 years of age (A2), ileocolonic disease (L3) was the most common location, and behavior was most often non-stricturing, non-penetrating (B1) or stricturing (B2). Approximately one-third had perianal disease. Among patients with UC, disease extent was distributed across proctitis (E1), left-sided colitis (E2), and extensive colitis (E3). At the time of assessment, most patients with UC had moderate clinical activity (Mayo score S2).

### 3.2. Treatment History and Laboratory Parameters

Treatment history and laboratory findings are summarized in Table 2. Just over half of the cohort (143/271; 52.8%) had ever received 5-aminosalicylates. Among 147 patients with available corticosteroid data, 3 (2.0%) had received intravenous steroids only, 107 (72.8%) had received oral steroids only, and 37 (25.2%) had received both. Of 141 patients with evaluable steroid response, 132 (93.6%) were steroid-responsive, and 9 (6.4%) were steroid-dependent. Data on biologic therapy showed that 160 patients (62.3%) had received at least one biologic agent, whereas 97 (37.7%) had never received biologics. Regarding steroid use, the dataset captured route of administration (oral, intravenous, or both) and steroid response (responsive vs. dependent) but did not distinguish between current and prior steroid use. Among 148 patients with available steroid data, 101 (68.2%) had received oral steroids only, 32 (21.6%) had received both oral and intravenous steroids, and 3 (2.0%) had received intravenous steroids only. Of 141 patients with documented response, 132 (93.6%) were steroid-responsive and 9 (6.4%) were steroid-dependent. Given that steroid use likely reflects prior or intermittent disease activity rather than current exposure, these data should be interpreted as indicators of disease course severity rather than contemporaneous treatment status. Similarly, biologic therapy data reflect cumulative exposure; current biologic use at the time of assessment was not separately recorded.

Mean hemoglobin was 12.6 (2.2) g/dL. Mean ESR and CRP were 21.8 (22.2) mm/hr and 19.1 (37.8) mg/L, respectively. Mean vitamin D concentration was 52.0 (39.0) nmol/L. Mean ferritin was 65.2 (132.8) ng/mL. Transferrin saturation was low in 47/271 patients (17.3%), normal in 222 (81.9%), and high in 2 (0.7%), indicating a notable proportion with possible functional iron deficiency (Table 2).

### 3.3. Prevalence and Severity of Fatigue

Based on BFI-A categories, 61 patients (21.3%) reported no fatigue, 66 (23.1%) mild fatigue, 104 (36.4%) moderate fatigue, and 55 (19.2%) severe fatigue (Table 3, Table 4 and Table 5). Overall, 159 of 286 patients (55.6%) reported moderate-to-severe fatigue.

### 3.4. Fatigue and Demographic, Lifestyle, and Comorbidity Variables

Associations between fatigue category and demographic, lifestyle, and comorbidity variables are shown in Table 3. There were no significant differences across fatigue levels in age (*p* = 0.062), sex (*p* = 0.780), BMI (*p* = 0.100), or smoking status (*p* = 0.331). Patients without fatigue had a slightly higher median age (32.0 years) than those with fatigue (27.0–29.0 years), but the difference was not statistically significant. Given the inclusion of adolescent patients (age 12–17 years; n = 3, 1.1% of cohort), we performed a supplementary analysis stratifying patients into age groups (12–17 years, 18–29 years, 30–44 years, and ≥45 years). Fatigue distribution did not differ significantly across age groups (*p* > 0.05), though the adolescent subgroup was too small for meaningful inference. Puberty-related hormonal changes may independently contribute to fatigue in younger patients; however, the very small number of adolescents in our cohort precludes subgroup-specific conclusions, and this should be considered a limitation of the age range applied.

Neither the presence of any comorbidity (*p* = 0.923) nor individual comorbidities, including diabetes (*p* = 1.000), hypertension (*p* = 0.645), thyroid disease (*p* = 0.731), and connective tissue disease (*p* = 0.889), differed significantly between fatigue categories (Table 3). Regarding occupational and lifestyle factors, employment and student status were captured in our dataset. Among 177 patients with available data, approximately 118 (66.7%) were employed, 39 (22.0%) were students, and 31 (17.5%) were unemployed. Formal assessment of physical activity levels (e.g., exercise frequency or validated physical activity measures) was not conducted in this study. While occupational status did not significantly predict fatigue category in exploratory analysis, the absence of objective physical activity and sedentary behavior data represents a limitation, given that physical activity is an important modifiable contributor to fatigue in IBD.

### 3.5. Fatigue, Treatment Exposure, and Laboratory Markers

Associations between fatigue severity, treatment history, and laboratory values are presented in Table 4. Exposure to 5-aminosalicylates did not differ across fatigue categories (*p* = 0.455). Likewise, neither the route of corticosteroid administration (*p* = 0.326) nor steroid responsiveness (*p* = 0.854) was associated with fatigue severity. Biologic exposure also did not differ significantly between fatigue categories (*p* = 0.163).

Most laboratory parameters, including hemoglobin, platelet distribution width, ferritin, TSH, CRP, vitamin D, and transferrin saturation, did not differ significantly across fatigue categories (all *p* > 0.10). Two measures showed statistically significant differences: serum iron (*p* = 0.011) and ESR (*p* = 0.023). Median serum iron tended to be higher in the moderate and severe fatigue groups than in the no-fatigue group, whereas ESR was highest in the no-fatigue group. However, neither parameter showed a clear monotonic pattern across fatigue levels (Table 4).

To evaluate independent predictors of clinically significant fatigue, a multivariable logistic regression model was performed with moderate-to-severe fatigue as the dependent variable.

In the adjusted model, serum iron was the only variable independently associated with moderate-to-severe fatigue (OR = 1.13, 95% CI 1.00–1.27, *p* = 0.042). Age, sex, comorbidity status, 5-ASA exposure, ESR, and vitamin D level were not significantly associated with fatigue status.

Overall model performance was modest (Pseudo R^2^ = 0.08; AUC = 0.67), indicating limited explanatory power of routine clinical variables for fatigue in this cohort (Table 5).

### 3.6. Fatigue and Disease Phenotype (Montreal Classification)

Table 6 summarizes associations between fatigue and disease phenotype. Overall, IBD diagnosis (CD, UC, IBD-unclassified) was not significantly associated with fatigue category (*p* = 0.478). Among patients with CD, age at diagnosis (A1–A3; *p* = 0.289), disease location (L1–L3; *p* = 0.660), upper gastrointestinal involvement (L4; *p* = 0.327), disease behavior (B1–B3; *p* = 0.894), and perianal disease (*p* = 0.888) were not associated with fatigue severity.

In UC, neither disease extent (E1–E3; *p* = 0.704) nor Mayo score (S0–S3; *p* = 0.662) differed significantly across fatigue categories (Table 6).

## 4. Discussion

### 4.1. Prevalence of Fatigue and Comparison with International Data

In this tertiary-care Saudi cohort, just over half of patients (55.6%) reported moderate-to-severe fatigue on the BFI-A, despite a relatively young mean age and few medical comorbidities. This prevalence is comparable to that reported in other regions. Population- and clinic-based studies from Europe and North America generally estimate clinically significant fatigue in about 40–60% of adults with IBD, rising to 70–80% among those with active disease, and remaining around 30–50% even when disease is considered quiescent [4,5,6,7,8,14,19,20].

Studies using other validated fatigue instruments have reported similar patterns. Spanish and Norwegian outpatient cohorts, for example, found fatigue in 41–54% of patients and “substantial fatigue” in roughly half, while cohorts restricted to patients in remission still reported fatigue in around 33–40% of cases [15,16,17,18]. The IBSEN III inception cohort in Norway further showed that substantial fatigue is present at diagnosis in approximately 60–70% of patients and that a considerable proportion continue to report chronic fatigue one year later, even when endoscopic and histologic remission has been achieved [19,20].

Data from outside Europe and North America are broadly consistent. Studies from Korea, China, and New Zealand reported fatigue in approximately 40–60% of unselected outpatients and in up to 80% of those with active disease, with a clear residual burden in clinical remission [21,22,23,24]. Latin American studies, including Mexican patients assessed using the IBD-F scale, reported fatigue in more than 80% of participants [25].

Taken together, these findings suggest that the 55.6% prevalence of moderate-to-severe fatigue in our cohort falls within the internationally reported range and aligns with the limited emerging evidence from Middle Eastern IBD populations [3,7,26].

### 4.2. Demographic, Disease-Related, and Laboratory Correlates

In our cohort, fatigue severity was not associated with age, sex, BMI, smoking status, or comorbidities. Evidence on demographic predictors of fatigue in IBD is mixed. Some studies, including work in clinically inactive Spanish outpatients, have reported higher fatigue in women and in those with higher BMI [15]. In contrast, other cohorts, particularly from Asian and Scandinavian settings, have not confirmed strong independent effects of age or sex after accounting for mood and sleep [17,19,21,22,23]. A recent meta-analysis concluded that demographic factors show inconsistent and generally modest associations with fatigue [7]. Our findings are consistent with this conclusion and may partly reflect the low burden of age-related and cardiometabolic disease in this relatively young sample.

We also found no association between fatigue and IBD subtype, Montreal phenotype, or Mayo score for UC. Most contemporary studies do not identify consistent differences in fatigue between CD and UC after accounting for other variables [4,5,7,8,15,16,17,18,19,20,21,22,23]. Montreal classification features (age at diagnosis, location, behavior, and extent) rarely emerge as independent predictors in multivariable models [15,16,17,21,22,23]. Clinical activity indices often show higher fatigue scores in patients with active disease in unadjusted comparisons, but these associations are frequently attenuated or lost once depression, anxiety, and sleep disturbance are included [13,15,16,17,18,21,22,23,30]. Regarding the link between fatigue and disease activity, it is important to note that our cohort did not exclude patients with active disease, and Mayo scores were available for UC patients. Among UC patients with severe disease (S3, n = 25), fatigue distribution did not differ significantly from those with mild or moderate activity (*p* = 0.662), consistent with evidence that disease activity alone is an insufficient predictor of fatigue. The absence of a validated CD activity score and the lack of contemporaneous endoscopic assessment in all patients remain limitations, as subclinical inflammation cannot be fully excluded. This is discussed further in Section 4.5.

In the IBSEN III cohort, Holten and colleagues reported correlations between both clinical and endoscopic activity and fatigue at diagnosis, particularly in UC. However, depressive symptoms, pain, and sleep disturbance were stronger independent correlates in both CD and UC [19], and chronic fatigue remained common at one year, even among patients in remission [20]. Together with our results, these findings suggest that conventional clinical activity measures do not fully account for fatigue, particularly in tertiary-care cohorts where many patients meet biochemical or clinical targets yet continue to experience persistent symptoms and psychosocial stressors [3,4,8,13].

Although iron deficiency was present in a subset of our patients, we did not detect significant associations between fatigue severity and hemoglobin, ferritin, transferrin saturation, CRP, or vitamin D levels. These results align with earlier work showing that chronic fatigue is more common in IBD than in controls but is only weakly associated with routine laboratory tests [4,5,6,8,17]. In several cohorts, anemia and elevated inflammatory markers were associated with fatigue in univariate analyses but became less prominent after accounting for mood and sleep [15,16,17,18,21,22,23].

For example, Yoo et al. reported that anemia and elevated ESR were associated with fatigue in Korean patients with UC but not in those with CD [21]. In a Chinese UC cohort, Xu et al. found that anemia predicted fatigue in unadjusted analyses but did not remain significant after adjustment for anxiety, depression, and disease activity [22]. In a New Zealand study using BFI/MFI, Aluzaite et al. reported that iron deficiency was not associated with fatigue [23]. Norwegian data from Frigstad et al. showed that clinical activity, depressive symptoms, and sleep disturbance, rather than CRP, fecal calprotectin, or vitamin D, were independently associated with chronic fatigue [17].

Overall, our negative findings for vitamin D and standard iron indices support the view that routine hematological and biochemical measures explain only a small proportion of fatigue variability in IBD [4,5,6,7,8,17,21,22,23]. The statistically significant differences in serum iron and ESR across fatigue categories did not follow a convincing dose–response pattern (e.g., serum iron was lowest and ESR was highest in the no-fatigue group). Given missing data and multiple comparisons, these signals are more plausibly due to chance than to a biologically meaningful relationship.

### 4.3. Treatment Exposure

We did not observe clear associations between fatigue severity and 5-ASA use, corticosteroid exposure or response, or ever-use of biologic therapy. Evidence for drug-specific effects on fatigue remains limited and difficult to interpret due to confounding by indication [7,8,9,12]. Corticosteroids can worsen sleep, mood, and muscle strength, potentially exacerbating fatigue, particularly with prolonged use. However, in observational cohorts, steroid exposure often reflects more severe disease, complicating causal inference [6,7,8,10,12].

Biologic therapies and other advanced agents often improve fatigue, alongside symptoms and inflammatory markers, in trials and observational studies using the FACIT-F and related tools [7,8,13,15,16,17,18,24]. Even so, many biologic-treated patients report clinically relevant fatigue despite good disease control [9,31,32], and neither trough levels nor anti-drug antibodies reliably distinguish fatigued from non-fatigued patients [33]. Across studies, specific medication classes rarely remain strong independent predictors after controlling for disease activity, mood, and sleep [7,8,9,13,15,16,17,18,22,23,24,30,33,34].

Our study was not designed to evaluate medication effects in detail. Exposure was coded as ever versus never, and we did not systematically capture objective treatment response measures (e.g., fecal calprotectin, contemporaneous endoscopy, or drug levels). Within these constraints, the lack of a clear association between simple medication exposure and fatigue is consistent with the broader literature and supports the notion that escalation of anti-inflammatory therapy alone is unlikely to resolve fatigue in patients who already meet conventional treatment targets [7,8,13,14,33,34].

### 4.4. Psychosocial and Behavioral Contributors

A key consideration in interpreting our findings is that we did not collect standardized measures of depression, anxiety, sleep disturbance, pain, physical activity, or HRQoL. A substantial body of evidence indicates that these psychosocial and behavioral factors are among the most consistent correlates of fatigue in IBD [3,4,7,8,9,10,13,15,16,17,18,21,22,23,24,25,30,31,32,33,34]. Access to psychiatric medication history (including antidepressants, benzodiazepines, and sleep aids) was not systematically available from the electronic record for all patients. Antidepressant use and comorbid psychiatric diagnoses may independently contribute to or mask fatigue, and their absence represents a further limitation of this dataset. Future studies should prospectively capture psychiatric diagnoses, psychotropic medication use, and validated mood and anxiety scores.

Multicenter cohorts from Spain, Scandinavia, Asia, Oceania, and Latin America have shown that depressive symptoms, anxiety, and poor sleep quality are strong independent predictors of fatigue, often with larger effect sizes than disease activity, hemoglobin, or CRP [13,15,16,17,18,21,22,23,25,30,32]. In clinically inactive Spanish outpatients, Villòria et al. found that depression scores and lower HRQoL remained key independent predictors of fatigue [15]. Chavarría et al. similarly reported associations between anxiety, depression, extraintestinal manifestations, poor sleep quality, and higher fatigue levels [16]. Frigstad et al. showed that depressive symptoms and sleep disturbance, but not vitamin D or objective inflammation, were independently related to chronic fatigue [17], and Mexican IBD-F data highlighted sleep disturbance, longer sleep latency, and depression as important contributors [25].

More detailed analyses clarify the interplay among disease activity, mood, sleep, and fatigue. Structural equation models from Australian cohorts indicate that, particularly in CD, the apparent effect of IBD activity on fatigue is largely indirect and mediated by depression, anxiety, and sleep disturbance [30]. Latent profile analyses have identified distinct fatigue profiles characterized by combinations of high fatigue, poor mental health, sleep problems, and active disease, with female sex, obesity, and opioid use more common in the highest-burden profiles [31]. Longitudinal data from IBSEN III, IBD Partners, and other cohorts suggest that within-person changes in depression, anxiety, sleep, and perceived stress often track more closely with changes in fatigue than changes in clinical activity alone [19,20,32,34].

Within this framework, it is not surprising that we observed few robust associations between fatigue and demographic, disease-related, or laboratory factors. Unmeasured psychosocial and behavioral variables likely explain a substantial portion of the variance in BFI-A scores in our cohort. This should not be interpreted as indicating that inflammation, anemia, or micronutrient abnormalities are unimportant; rather, these factors likely operate within a broader biopsychosocial context that includes mood, sleep, pain, and daily functioning [7,8,10,12,13,14,30,31,32,33,34]. Future studies in Saudi Arabia and the wider region should incorporate validated Arabic-language measures of depression and anxiety, sleep quality, pain interference, physical activity, and IBD-specific HRQoL to better characterize determinants of fatigue and inform targeted interventions [3,4,7,8,9,13,15,16,17,18,21,22,23,24,25,30,31,32,33,34].

### 4.5. Strengths and Limitations

This study has several strengths. We used a validated Arabic version of a dedicated fatigue instrument (BFI-A) [27], collected clinical and laboratory data in a standardized manner, and included a moderately large sample from a tertiary IBD service. The cohort was relatively young and had few major comorbidities, reducing the likelihood that age-related cardiovascular or metabolic conditions accounted for fatigue and helping to more specifically characterize fatigue in relation to IBD.

Several limitations should, nonetheless, be acknowledged. The cross-sectional design precludes causal inference and does not distinguish transient from persistent fatigue. As a single-center tertiary-care study, generalizability to community settings may be limited, as tertiary clinics often manage more complex or treatment-refractory disease and have higher biologic exposure. We did not assess key psychosocial and behavioral domains despite strong evidence that they are central to fatigue in IBD [3,4,7,8,9,13,15,16,17,18,21,22,23,24,25,30,31,32,33,34]. Disease activity was assessed using clinical indices and routine blood markers rather than standardized fecal calprotectin or contemporaneous endoscopy for all patients; therefore, residual or unrecognized inflammatory activity cannot be excluded. Finally, some laboratory variables had substantial missing data, and the number of statistical comparisons increases the risk of chance findings. Additionally, data on prior bowel resection or ostomy formation were not systematically captured in this dataset. Patients who have undergone intestinal surgery, including those with a stoma, may experience fatigue through distinct mechanisms related to nutritional deficiency, altered gut anatomy, and psychological adjustment, and this subgroup should be characterized separately in future studies.

## 5. Conclusions

Fatigue is frequently observed in Saudi patients with IBD, yet it often goes undetected through standard history-taking, physical exams, or routine lab tests, making direct assessment crucial. In this group from a tertiary-care setting, over half of the patients experienced moderate-to-severe fatigue. The severity of fatigue showed no correlation with gender, body mass index, smoking habits, IBD type, or exposure to corticosteroids or biologics, nor with standard hematologic and biochemical indicators. These results are consistent with global data, reinforcing the notion that fatigue related to IBD is a somewhat distinct symptom that traditional biomedical markers fail to adequately capture. Implementing regular fatigue assessments using concise, validated tools like the BFI-A, along with evaluations for mood and sleep disturbances and structured inquiries about pain and physical activity, could help identify patients in need of multidisciplinary care who might otherwise be missed. Long-term, multicenter research incorporating biological, psychological, and behavioral metrics is necessary to identify high-risk groups and evaluate multimodal strategies to mitigate this debilitating symptom. Future studies should prospectively examine the correlation between fatigue severity and objective disease activity (including endoscopic and biomarker-confirmed remission), current treatment regimen (with distinction between past and current biologic or steroid exposure), and standardized measures of anxiety and depression. Such integrated analyses, stratified by fatigue grade, will be essential to identify high-risk subgroups and develop targeted, multimodal interventions for IBD-related fatigue in this population.

## Figures and Tables

**Table 1:** Baseline demographic and clinical characteristics of the study cohort.

Characteristic	Overall, N = 286
Age, years	
Mean (SD)	30.8 (9.1)
Range	12–66
Sex, n (%)	
Female	121 (42.3%)
Male	165 (57.7%)
Anthropometrics, mean (SD)	
Weight, kg	64.5 (19.8)
Height, cm	163.4 (11.6)
BMI, kg/m^2 1^	23.8 (6.0)
Smoking status, n (%)	
Current smoker	32 (11.2%)
Former smoker	10 (3.5%)
Never smoker	244 (85.3%)
Comorbidities, n (%)	
≥1 comorbidity ^2^	40 (14.4%)
None ^2^	238 (85.6%)
Diabetes ^3^	3 (1.1%)
Hypertension ^3^	5 (1.9%)
Thyroid disorder ^3^	7 (2.6%)
Connective tissue disease ^3^	3 (1.1%)

^1^ Missing data for 1 patient (BMI). ^2^ Missing data for 8 patients (comorbidity status). ^3^ Missing data for 16 patients (individual comorbidities). Percentages are calculated using available data (excluding missing values). BMI, body mass index; SD, standard deviation.

**Table 2:** Laboratory parameters and treatment history of the study cohort.

Treatment, n (%)	
5-ASA Use ^1^	
Ever	143 (52.8%)
Never	128 (47.2%)
Steroid administration, n (%) ^2^	
Intravenous (IV)	3 (2.0%)
Oral (PO)	107 (72.8%)
Both PO and IV	37 (25.2%)
Steroid response, n (%) ^3^	
Steroid-responsive	132 (93.6%)
Steroid-dependent	9 (6.4%)
Biologic therapy use, n (%) ^4^	
Ever	160 (62.3%)
Never	97 (37.7%)
Laboratory values, mean (SD)	
Hemoglobin, g/dL ^5^	12.6 (2.2)
Platelet distribution width, fL ^6^	12.3 (7.7)
Ferritin, ng/mL ^7^	65.2 (132.8)
TSH, mIU/L ^8^	2.8 (3.6)
Erythrocyte sedimentation rate, mm/hr ^9^	21.8 (22.2)
C-reactive protein, mg/L ^10^	19.1 (37.8)
Vitamin D, nmol/L ^11^	52.0 (39.0)
Transferrin saturation, n (%) ^12^	
Normal	222 (81.9%)
Low	47 (17.3%)
High	2 (0.7%)

^1^ Missing data for 15 patients (5-ASA use; transferrin saturation). ^2^ Missing data for 139 patients (steroid administration). ^3^ Missing data for 145 patients (steroid response). ^4^ Missing data for 29 patients (biologic therapy use; n = 257 available). ^5^ Missing data for 25 patients (hemoglobin). ^6^ Missing data for 43 patients (PDW). ^7^ Missing data for 91 patients (ferritin). ^8^ Missing data for 219 patients (TSH). ^9^ Missing data for 82 patients (ESR). ^10^ Missing data for 50 patients (CRP). ^11^ Missing data for 121 patients (vitamin D). 5-ASA, 5-aminosalicylate; SD, standard deviation; TSH, thyroid-stimulating hormone. CRP > 5 mg/L is considered elevated. Exposure to steroid include any historical exposure, not just exposure at the time of study. ^12^ Missing data for 15 patients (Transferrin saturation).

**Table 3:** Association between fatigue severity and demographic, lifestyle, and comorbidity profiles.

Characteristic	No Fatigue (n = 61)	Mild Fatigue (n = 66)	Moderate Fatigue (n = 104)	Severe Fatigue (n = 55)	*p*-Value
Age, years					0.062
Median (IQR)	32.0 (26.0–38.0)	27.0 (22.2–33.0)	29.0 (24.0–36.2)	28.0 (25.0–36.0)	
Sex, n (%)					0.780
Female	28 (45.9%)	29 (43.9%)	40 (38.5%)	24 (43.6%)	
Male	33 (54.1%)	37 (56.1%)	64 (61.5%)	31 (56.4%)	
BMI, kg/m^2^					0.100
Median (IQR)	22.2 (18.7–25.3)	22.9 (20.9–25.3)	24.2 (20.2–26.7)	24.3 (19.5–28.6)	
Smoking status, n (%)					0.331
Current smoker	9 (14.8%)	3 (4.5%)	15 (14.4%)	5 (9.1%)	
Former smoker	1 (1.6%)	3 (4.5%)	3 (2.9%)	3 (5.5%)	
Never smoker	51 (83.6%)	60 (90.9%)	86 (82.7%)	47 (85.5%)	
≥1 comorbidity, n (%)					0.923
No	51 (86.4%)	57 (87.7%)	85 (85.0%)	45 (83.3%)	
Yes	8 (13.6%)	8 (12.3%)	15 (15.0%)	9 (16.7%)	
Diabetes, n (%)					1.000
No	56 (98.2%)	63 (98.4%)	98 (99.0%)	50 (100.0%)	
Yes	1 (1.8%)	1 (1.6%)	1 (1.0%)	0 (0.0%)	
Hypertension, n (%)					0.645
No	57 (100.0%)	62 (96.9%)	97 (98.0%)	49 (98.0%)	
Yes	0 (0.0%)	2 (3.1%)	2 (2.0%)	1 (2.0%)	
Thyroid disorder, n (%)					0.731
No	56 (98.2%)	61 (95.3%)	97 (98.0%)	49 (98.0%)	
Yes	1 (1.8%)	3 (4.7%)	2 (2.0%)	1 (2.0%)	
Connective tissue disease, n (%)					0.889
No	57 (100.0%)	63 (98.4%)	98 (99.0%)	49 (98.0%)	
Yes	0 (0.0%)	1 (1.6%)	1 (1.0%)	1 (2.0%)	

Note: Denominators vary due to missing data; thus, totals within categories may not sum to the group total. BMI, body mass index; IQR, interquartile range.

**Table 4:** Association between fatigue severity and treatment, response, and laboratory parameters.

Parameter	No Fatigue (n = 61)	Mild Fatigue (n = 66)	Moderate Fatigue (n = 104)	Severe Fatigue (n = 55)	*p*-Value
5-ASA use					0.455
Ever	36 (61.0%)	34 (54.0%)	48 (48.0%)	25 (51.0%)	
Never	23 (39.0%)	29 (46.0%)	52 (52.0%)	24 (49.0%)	
Steroid administration					0.326
IV	2 (6.1%)	1 (3.4%)	0 (0.0%)	0 (0.0%)	
PO	24 (72.7%)	18 (62.1%)	41 (78.8%)	24 (72.7%)	
PO, IV	7 (21.2%)	10 (34.5%)	11 (21.2%)	9 (27.3%)	
Steroid response					0.854
Steroid-dependent	3 (9.1%)	2 (6.9%)	2 (4.3%)	2 (6.2%)	
Steroid-responsive	30 (90.9%)	27 (93.1%)	45 (95.7%)	30 (93.8%)	
Biologic therapy use					0.163
Ever	16 (50%)	70 (70%)	59 (59%)	32 (58.2%)	
Never	16 (50%)	30 (30%)	41 (41%)	21 (41.8%)	
Hemoglobin, g/dL					0.545
Median (IQR)	13.0 (11.2–14.1)	12.4 (11.1–14.2)	13.1 (11.0–14.5)	12.3 (11.3–13.7)	
PDW, fL					0.116
Median (IQR)	11.4 (10.5–13.4)	11.4 (9.9–13.1)	11.8 (10.6–13.6)	10.9 (9.7–12.2)	
Ferritin, ng/mL					0.857
Median (IQR)	36.9 (11.4–68.5)	33.9 (8.1–89.8)	27.5 (10.4–68.2)	26.5 (8.6–54.9)	
Serum Iron, µg/dL					0.011
Median (IQR)	4.2 (2.3–6.4)	4.4 (2.4–10.8)	7.0 (4.0–13.6)	8.1 (4.4–11.7)	
TSH, mIU/L					0.439
Median (IQR)	1.5 (1.2–2.5)	2.2 (1.4–3.5)	2.1 (1.5–3.4)	2.2 (1.4–3.8)	
ESR, mm/hr					0.023
Median (IQR)	22.0 (8.0–38.0)	14.5 (6.0–31.2)	12.0 (4.2–19.8)	18.0 (8.0–32.0)	
CRP, mg/L					0.143
Median (IQR)	5.8 (3.2–21.9)	3.4 (3.2–12.6)	3.5 (3.1–12.8)	3.3 (3.2–12.2)	
Vitamin D, nmol/L					0.300
Median (IQR)	38.0 (25.9–59.5)	40.2 (24.3–68.4)	47.0 (30.4–69.2)	41.5 (25.0–56.0)	
Transferrin saturation, n (%)					0.785
Normal	48 (80.0%)	51 (81.0%)	82 (83.7%)	41 (82.0%)	
Low	12 (20.0%)	11 (17.5%)	16 (16.3%)	8 (16.0%)	
High	0 (0.0%)	1 (1.6%)	0 (0.0%)	1 (2.0%)	

Note: Denominators vary due to missing data; thus, totals within categories may not sum to the group total. 5-ASA, 5-aminosalicylate; CRP, C-reactive protein; ESR, erythrocyte sedimentation rate; IQR, interquartile range; IV, intravenous; PDW, platelet distribution width; PO, oral; TSH, thyroid-stimulating hormone.

**Table 5:** Multivariable logistic regression analysis of factors associated with moderate-to-severe fatigue. Sample size: n = 286; Dependent variable: BFI; Independent variables: 7.

Variable	Coefficient (B)	SE	OR	95% CI	*p*-Value
Intercept	−0.008	1.159	N/A	N/A	0.9942
Age	−0.039	0.026	0.96	[0.91, 1.01]	0.1384
Gender (Female vs. Male)	−0.145	0.523	0.86	[0.31, 2.41]	0.7817
Comorbidity (Yes vs. No)	0.518	0.735	1.68	[0.40, 7.09]	0.4806
5ASA (Ever vs. Never)	0.450	0.527	1.57	[0.56, 4.41]	0.3928
serum iron	0.120	0.059	1.13	[1.00, 1.27]	0.0421
ESR	0.001	0.014	1.00	[0.97, 1.03]	0.9258
Vitamin D level	0.007	0.008	1.01	[0.99, 1.02]	0.3478

Log-Likelihood: −49.20, AIC: 114.40, Pseudo R^2^: 0.08. Accuracy: 0.59, Precision: 0.61, AUC: 0.67. Note. B = logistic regression coefficient; OR = odds ratio; SE = standard error; CI = confidence interval. Statistical significance was set at *p* < 0.05.

**Table 6:** Association between fatigue severity and disease phenotype (Montreal classification).

Disease Characteristic	No Fatigue (n = 61)	Mild Fatigue (n = 66)	Moderate Fatigue (n = 104)	Severe Fatigue (n = 55)	*p*-Value
Diagnosis					0.478
Ulcerative colitis	24 (39.3%)	18 (27.3%)	34 (32.7%)	18 (32.7%)	
Crohn’s disease	37 (60.7%)	47 (71.2%)	65 (62.5%)	35 (63.6%)	
IBD-U	0 (0.0%)	1 (1.5%)	5 (4.8%)	2 (3.6%)	
CD: Age at diagnosis (A) ^1^					0.289
A1 (≤16 years)	8 (21.6%)	18 (38.3%)	20 (30.8%)	8 (23.5%)	
A2 (17–40 years)	28 (75.7%)	27 (57.4%)	45 (69.2%)	25 (73.5%)	
A3 (>40 years)	1 (2.7%)	2 (4.3%)	0 (0.0%)	1 (2.9%)	
CD: Location (L) ^1^					0.660
L1 (ileal)	9 (24.3%)	12 (25.5%)	16 (25.0%)	11 (33.3%)	
L2 (colonic)	4 (10.8%)	10 (21.3%)	7 (10.9%)	3 (9.1%)	
L3 (ileocolonic)	24 (64.9%)	25 (53.2%)	41 (64.1%)	19 (57.6%)	
CD: Upper GI involvement ^1^					0.327
L4 (Yes)	4 (11.4%)	1 (2.1%)	5 (8.2%)	3 (9.1%)	
No	28 (80.0%)	35 (74.5%)	46 (75.4%)	27 (81.8%)	
CD: behavior (B) ^1^					0.894
B1 (non-stricturing, non-penetrating)	16 (43.2%)	24 (51.1%)	25 (39.1%)	17 (50.0%)	
B2 (stricturing)	11 (29.7%)	13 (27.7%)	23 (35.9%)	9 (26.5%)	
B3 (penetrating)	10 (27.0%)	10 (21.3%)	16 (25.0%)	8 (23.5%)	
CD: perianal disease ^1^					0.888
Yes	11 (31.4%)	17 (36.2%)	25 (39.1%)	13 (39.4%)	
No	24 (68.6%)	30 (63.8%)	39 (60.9%)	20 (60.6%)	
UC: extent (E) ^2^					0.704
E1 (proctitis)	5 (20.8%)	3 (16.7%)	8 (23.5%)	4 (22.2%)	
E2 (left-sided)	8 (33.3%)	9 (50.0%)	16 (47.1%)	5 (27.8%)	
E3 (extensive)	11 (45.8%)	6 (33.3%)	10 (29.4%)	9 (50.0%)	
UC: Mayo score ^2^					0.662
S0 (remission)	2 (8.3%)	1 (5.6%)	1 (2.9%)	1 (5.6%)	
S1 (mild)	3 (12.5%)	2 (11.1%)	9 (26.5%)	3 (16.7%)	
S2 (moderate)	11 (45.8%)	9 (50.0%)	19 (55.9%)	8 (44.4%)	
S3 (severe)	8 (33.3%)	6 (33.3%)	5 (14.7%)	6 (33.3%)	

^1^ Applied to patients with Crohn’s disease only (n = 184). ^2^ Applies to Ulcerative Colitis patients only (n = 94). CD, Crohn’s disease; GI, gastrointestinal; IBD-U, inflammatory bowel disease–unclassified; UC, ulcerative colitis. Denominators vary due to missing data; thus, totals within categories may not sum to the group n.

## Data Availability

Data supporting the conclusions of this article will be made available by the authors on request.

## Abbreviations

The following abbreviations are used in this manuscript:

5-ASA	5-aminosalicylate
BFI-A	Brief Fatigue Inventory–Arabic
BMI	body mass index
CBC	complete blood count
CD	Crohn’s disease
CRP	C-reactive protein
ESR	erythrocyte sedimentation rate
GI	gastrointestinal
HRQoL	health-related quality of life
IBD	inflammatory bowel disease
IQR	interquartile range
IV	intravenous
PO	per os (oral)
SD	standard deviation
TSH	thyroid-stimulating hormone
UC	ulcerative colitis
